# Association Between Pfizer COVID-19 Vaccine Adverse Effects and Diabetes Mellitus: A Prospective Multicenter Study

**DOI:** 10.7759/cureus.48263

**Published:** 2023-11-04

**Authors:** Syed Mamoon Akhtar, Zohair J Gazzaz, Mukhtiar Baig, Rabika Majeed, Atif A Hashmi

**Affiliations:** 1 Internal Medicine, Tawam Hospital, Abu Dhabi, ARE; 2 Internal Medicine, Faculty of Medicine in Rabigh, King Abdulaziz University, Jeddah, SAU; 3 Clinical Biochemistry, Faculty of Medicine in Rabigh, King Abdulaziz University, Jeddah, SAU; 4 Family Medicine, Al-Samdah Health Center Duba, Ministry of Health, Duba, SAU; 5 Pathology, Liaquat National Hospital and Medical College, Karachi, PAK

**Keywords:** fever, swelling, pain, covid-19, pfizer vaccine

## Abstract

Introduction

The epidemic of severe acute respiratory syndrome coronavirus 2 (SARS-CoV-2) triggered the contagion of coronavirus disease 2019 (COVID-19), which killed many individuals globally. The Pfizer BioNTech vaccine was the first messenger ribonucleic acid (mRNA)-based vaccine that boosted immunity against various adverse reactions. The objective of this study was to evaluate the frequency of Pfizer vaccine side effects among participants with and without diabetes mellitus (DM).

Methods

This multicenter study was cross-sectional and was performed using a non-probability consecutive sampling technique. The study duration was six months, from October 1, 2022, to March 31, 2023. A total of 750 participants who received both doses of the Pfizer vaccine were included in the study. Demographic details such as gender, age, comorbidities, preceding COVID-19 infection, and the occurrence of any local and systemic side effects of the first and second doses of vaccine were recorded. The association between local and general side effects and the presence of DM was assessed using the chi-square test.

Results

Of the 750 participants included in the study, 289 (77.1%) were males with diabetes mellitus (DM), and 217 (57.9%) were non-diabetic participants; however, 86 (22.9%) females had DM, and 158 (42.1%) were non-diabetic; their mean ages were 48.23 ± 16.22 and 37.56 ± 12.15 years, respectively. The most commonly occurring side effects after receiving the first dose of the Pfizer vaccine were: injection site burning in 251 (66.9%) diabetic and 254 (67.7%) non-diabetic participants. Likewise, the frequency of side effects of the second dose of the Pfizer vaccine showed that the most commonly reported side effects were: muscle pain, found in 240 (64.0%) diabetic patients and 194 (51.7%) non-diabetics, with a statistically significant association (p =0.001).

Conclusion

This study concluded that participants with DM had local and general adverse effects considerably more frequently than those without DM. The most frequently observed adverse effects in both diabetic and non-diabetic participants were injection site burning, rashes, muscle pain, and fever after receiving both doses of the Pfizer vaccine. Moreover, most of the side effects were minor.

## Introduction

The global epidemic of coronavirus disease 2019 (COVID-19) has killed millions of human beings and had drastic economic, social, and mental effects [1,2]. Massive global efforts are underway to create COVID-19 vaccinations, slow the progression of the pandemic, and save lives [3]. The only way to put an end to the immediate threat was to discover an effective vaccine, and in this regard, significant and swift progress has been made in vaccination research [4]. To combat the pandemic, AstraZeneca, Janssen (Johnson & Johnson), Sinovac, Sputnik V, Sinopharm, and Pfizer BioNTech were among the companies that produced COVID-19 vaccinations [5]. Although there are differences in the efficacy of these vaccinations in preventing COVID-19 infection, each type of immunization has distinctive benefits and drawbacks in terms of immunogenicity and efficacy [6].

The Pfizer/Biotech vaccine was the first to receive food and drug administration (FDA) approval for emergency use on December 11, 2020 [7]. The Pfizer-BioNTech Company has introduced several COVID-19 vaccine side effects and allergic responses. These include local responses, such as pain, swelling, and injection site redness, as well as systemic reactions, such as fever, chills, headache, nausea, vomiting, diarrhea, and sudden or more severe discomfort in the muscles and joints. Furthermore, there have been reports of serious adverse effects, including cerebrovascular events, acute myocardial infarction, hypersensitivity reactions, and appendicitis [8]. Initially, people hesitated to receive COVID-19 vaccines because of documented adverse effects and several misconceptions [9,10].

The wellbeing of the COVID-19 vaccination is crucial to ensuring that the advantages outweigh the risks. Nevertheless, phase 3 trials of the messenger ribonucleic acid (mRNA) vaccine may not detect severe or uncommon adverse effects because of the small sample size, inclusion requirements, and participant characteristics that might differ from those of the general population [11]. To identify rare and long-term side effects associated with vaccinations, the World Health Organization (WHO) recommended a post-marketing assessment of the safety profile of all vaccines [12].

The vaccines developed by BioNTech and Pfizer (BNT162b2) rely on mRNA technology. The spike (S) protein, a spike-like surface feature, is present in coronaviruses. Almost a billion people worldwide have received vaccinations made by Moderna, Pfizer BioNTech, Janssen, and Oxford-AstraZeneca that target the S protein of SARS-CoV-2 [13]. Numerous trials using a revolutionary technique known as mRNA technology are presently ongoing to produce vaccines [14].

In addition, post-marketing studies have shown little difference in the types and frequencies of adverse events reported by vaccine recipients [15]. With the increased global availability of vaccines, adverse effects should continue to be properly monitored. There is currently a dearth of information on the prevalence of chronic illnesses such as diabetes mellitus (DM) in resource-constrained countries such as Pakistan. Physicians should be aware of any possible side effects or issues with patients with DM before administering COVID-19 vaccines. Thus, the purpose of this study was to evaluate the frequency of both local and general side effects of Pfizer COVID-19 among diabetic and non-diabetic subjects in the population of Pakistan.

## Materials and methods

This was a cross-sectional study conducted at multiple centers in Karachi, Pakistan, using a non-probability consecutive sampling method. Ethical approval for the study was obtained from Essa General Hospital (Essa/74/2022). Informed written consent was obtained from all participants. All participants were given the choice to withdraw from the study at any time. This research was conducted over a six-month period, i.e., from October 1, 2022, to March 31, 2023. A total of 750 participants who received two doses of the Pfizer vaccine were included in the study. The age criterion for inclusion was 18 to 75 years. Participants who did not receive the Pfizer vaccine but received some other vaccine were excluded from the study. Severely immunocompromised patients were not included in the study; similarly, transplant recipients, patients with end-stage renal disease, patients undergoing dialysis, and those receiving chemotherapy for cancer were excluded from the study.

The participants’ demographic information was collected through a structured questionnaire at the time of vaccination. The demographic characteristics of the vaccinated subjects, including gender, age, and comorbidities, such as hypertension and DM, preceding exposure to COVID-19 contagion, were recorded. The duration of hypertension and DM was recorded. The total follow-up duration was six weeks to evaluate Pfizer vaccine side effects. The local and systemic adverse effects of the vaccine were evaluated at a one- to six-week interval after the administration of the Pfizer vaccine. The local side effects that were assessed included pain, redness, burning, and swelling at the injection site, whereas the systemic side effects recorded were fever, lymphadenopathy, headache, nausea, body rashes, flu-like illness, anxiety, myalgia, fatigue, joint pain, chills, cough, sore throat, dyspnea, diarrhea, and chest pain. The presence of side effects at any (one or six-week) point was recorded. Apart from this, the overall satisfaction level with the Pfizer vaccination was also asked of the participants and noted.

The data were entered and analyzed using IBM Corp. Released 2019. IBM SPSS Statistics for Windows, Version 26.0. Armonk, NY: IBM Corp. Age, height, weight, hypertension, and diabetes mellitus were expressed as the mean standard deviation. Demographic features, such as gender, frequency, and percentage of both local and general side effects, were documented. By applying an independent t-test, the relationship between the mean and standard deviation was determined. In addition, the association between local and general side effects for diabetic and non-diabetic patients and the overall satisfaction level of subjects receiving the first and second doses of Pfizer were assessed using the chi-square test. A p-value < 0.05 was considered statistically significant.

## Results

A total of 750 Pfizer vaccinated recipients who received first and second doses of Pfizer vaccination were studied; out of them, 289 (77.1%) were males with DM, and 217 (57.9%) were non-diabetic participants; however, 86 (22.9%) females had DM and 158 (42.1%) were non-diabetic; their mean ages were 48.23 ± 16.22 and 37.56 ± 12.15 years, respectively. The relationship between age and diabetic and non-diabetic participants was significant (p < 0.05). The mean weight was 71.66 ± 16.96 kg for diabetic and 62.66 ± 12.26 kg for non-diabetic participants, indicating a significant difference between them (p < 0.001). A mean height of 5.25 ± 0.61 feet for diabetics and 5.00 ± 0.60 feet for non-diabetics was observed, and a statistically significant difference was found between them (p < 0.001). The mean duration of DM was also observed at 6.88 ± 7.51 years. Among hypertensive patients, 307 (81.9%) had DM and 43 (11.5%) had non-diabetes, whereas among non-hypertensive patients, 68 (18.1%) were diabetic and 332 (88.5%) were non-diabetic; however, the relationship between both was statistically significant (p < 0.001). It was also noticed that out of 750 participants, only 39 (10.4%) diabetic patients had a preceding COVID-19 infection, as shown in Table 1.

**Table 1:** The demographic details of Pfizer-vaccinated participants (n=750) SD: standard deviation; COVID-19: coronavirus disease 2019 *significant as a p-value <0.05.

Variable		Mean±SD/n(%)		p-value
		Diabetes Mellitus		
		Yes	No	
Age (years)		48.23±16.22	37.56±12.15	<0.001*
Weight (kg)		71.66±16.96	62.66±12.26	<0.001*
Height (feet)		5.25±0.61	5.00±0.60	<0.001*
Hypertension, duration (years)		4.03±3.12	4.00±0.00	0.975
Gender	Male	289(77.1%)	217(57.9%)	<0.001*
	Female	86(22.9%)	158(42.1%)	
Hypertension	Yes	307(81.9%)	43(11.5%)	<0.001*
	No	68(18.1%)	332(88.5%)	
Previous COVID-19 infection	Yes	39(10.4%)	0(0.0%)	<0.001*
	No	336(89.6%)	375(100.0%)	

The most commonly occurring side effects found in the participants receiving the first dose of the Pfizer vaccine were: injection site burning 251 (66.9%) for diabetics and 254 (67.7%) for non-diabetics, with no significant relationship observed between them (p = 0.815). Additionally, muscle pain affected 248 (66.1%) diabetics and 153 (40.8%) non-diabetics, followed by fever in 242 (64.5%) diabetics and 283 (75.5%) non-diabetics, and injection site pain affected 235 (62.7%) diabetics and 190 (50.7%) non-diabetics, with a statistically significant difference between them (p < 0.05). Similarly, a significant difference (p < 0.05) was observed between the diabetic and non-diabetic participants for adverse effects, including lymphadenopathy, headache, joint pain, cough, sore throat, and diarrhea. An insignificant association (p > 0.05) was noted between the majority of diabetes and non-diabetes participants with the side effects of swelling at the injection site, redness (injection site), nausea, rashes, flu, anxiety, fatigue, chills, shortness of breath, and chest pain (Table 2).

**Table 2:** The incidence of side effects after first dose of Pfizer vaccine among diabetes and non-diabetes participants *p-value significant as a <0.05.

Variable		n (%)		p-value
		Diabetes mellitus		
		Yes	No	
Pain at the site of injection	Yes	235(62.7%)	190(50.7%)	0.001*
	No	140(37.3%)	185(49.3%)	
Swelling at the site of injection	Yes	183(48.8%)	191(50.9%)	0.559
	No	192(51.2%)	184(49.1%)	
Redness at the site of injection	Yes	53(14.1%)	56(14.9%)	0.756
	No	322(85.9%)	319(85.1%)	
Lymphadenopathy	Yes	158(42.1%)	95(25.3%)	<0.001*
	No	217(57.9%)	280(74.7%)	
Fever (temperature >37.8 ˚C)	Yes	242(64.5%)	283(75.5%)	0.001*
	No	133(35.5%)	92(24.5%)	
Headache	Yes	182(48.5%)	103(27.5%)	<0.001*
	No	193(51.5%)	272(72.5%)	
Nausea	Yes	56(14.9%)	62(16.5%)	0.547
	No	319(85.1%)	313(83.5%)	
Rashes	Yes	163(43.5%)	151(40.3%)	0.374
	No	212(56.5%)	224(59.7%)	
Burning at injection site	Yes	251(66.9%)	254(67.7%)	0.815
	No	124(33.1%)	121(32.3%)	
Flu-like illness	Yes	100(26.7%)	87(23.2%)	0.273
	No	275(73.3%)	288(76.8%)	
Anxiety	Yes	103(27.5%)	99(26.4%)	0.742
	No	272(72.5%)	276(73.6%)	
Myalgia	Yes	248(66.1%)	153(40.8%)	<0.001*
	No	127(33.9%)	222(59.2%)	
Fatigue	Yes	134(35.7%)	122(32.5%)	0.355
	No	241(64.3%)	253(67.5%)	
Joint pain	Yes	205(54.7%)	235(62.7%)	0.026*
	No	170(45.3%)	140(37.3%)	
Chills	Yes	154(41.1%)	159(42.4%)	0.711
	No	221(58.9%)	216(57.6%)	
Cough	Yes	165(44.0%)	87(23.2%)	<0.001*
	No	210(56.0%)	288(76.8%)	
Sore throat	Yes	159(42.4%)	194(51.7%)	0.010*
	No	216(57.6%)	181(48.3%)	
Shortness of breath	Yes	113(30.1%)	106(28.3%)	0.574
	No	262(69.9%)	269(71.7%)	
Diarrhea	Yes	142(37.9%)	115(30.7%)	0.038*
	No	233(62.1%)	260(69.3%)	
Chest pain	Yes	103(27.5%)	100(26.7%)	0.805
	No	272(72.5%)	275(73.3%)	

Likewise, the distribution of side effects of the second dose of the Pfizer vaccine showed that the most commonly reported side effects in recipients were: muscle pain, found in 240 (64.0%) diabetic patients and 194 (51.7%) in non-diabetics, with a statistically significant association between them (p = 0.001). Similarly, rashes were noticed in 226 (60.3%) diabetics and 198 (52.8%) non-diabetes participants, with a significant relationship between them (p = 0.039). For diabetics, injection site pain was 204 (54.4%) and 218 (58.1%) for non-diabetics, with an insignificant difference between these groups (p = 0.303). Correspondingly, a significant difference (p < 0.05) was observed between the majority of diabetic and non-diabetic participants for adverse effects of redness and swelling at the injection site, fever, nausea, burning (injection site), headache, flu, fatigue, joint pain, chills, sore throat, shortness of breath, diarrhea, and chest pain, while an insignificant association (p > 0.05) was noted between the few diabetes and non-diabetes participants against side effects like lymphadenopathy, anxiety, and cough, as shown in Table 3.

**Table 3:** The incidence of side effects after the second dose of the Pfizer vaccine among diabetes and non-diabetes participants *p-value is significant at <0.05.

Variable		n (%)		p-value
		Diabetes mellitus		
		Yes	No	
Pain at the site of injection	Yes	204(54.4%)	218(58.1%)	0.303
	No	171(45.6%)	157(41.9%)	
Swelling at the site of injection	Yes	173(46.1%)	212(56.5%)	0.004*
	No	202(53.9%)	163(43.5%)	
Redness at the site of injection	Yes	57(15.2%)	9(2.4%)	<0.001*
	No	318(84.8%)	366(97.6%)	
Lymphadenopathy	Yes	135(36.0%)	133(35.5%)	0.879
	No	240(64.0%)	242(64.5%)	
Fever (temperature > 37.8 ˚C)	Yes	173(46.1%)	138(36.8%)	0.009*
	No	202(53.9%)	237(63.2%)	
Headache	Yes	121(32.3%)	208(55.5%)	<0.001*
	No	254(67.7%)	167(44.5%)	
Nausea	Yes	57(15.2%)	24(6.4%)	<0.001*
	No	318(84.8%)	351(93.6%)	
Rashes	Yes	226(60.3%)	198(52.8%)	0.039*
	No	149(39.7%)	177(47.2%)	
Burning at injection site	Yes	166(44.3%)	210(56.0%)	0.001*
	No	209(55.7%)	165(44.0%)	
Flu	Yes	76(20.3%)	44(11.7%)	0.001*
	No	299(79.7%)	331(88.3%)	
Anxiety	Yes	160(42.7%)	140(37.3%)	0.136
	No	215(57.3%)	235(62.7%)	
Myalgia	Yes	240(64.0%)	194(51.7%)	0.001*
	No	135(36.0%)	181(48.3%)	
Fatigue	Yes	141(37.6%)	101(26.9%)	0.002*
	No	234(62.4%)	274(73.1%)	
Joint pain	Yes	187(49.9%)	138(36.8%)	<0.001*
	No	188(50.1%)	237(63.2%)	
Chills	Yes	130(34.7%)	185(49.3%)	<0.001*
	No	245(65.3%)	190(50.7%)	
Cough	Yes	81(21.6%)	87(23.2%)	0.599
	No	294(78.4%)	288(76.8%)	
Sore throat	Yes	62(16.5%)	118(31.5%)	<0.001*
	No	313(83.5%)	257(68.5%)	
Shortness of breath	Yes	146(38.9%)	197(52.5%)	<0.001*
	No	229(61.1%)	178(47.5%)	
Diarrhea	Yes	130(34.7%)	99(26.4%)	0.014*
	No	245(65.3%)	276(73.6%)	
Chest pain	Yes	157(41.9%)	114(30.4%)	0.001*
	No	218(58.1%)	261(69.6%)	

The overall satisfaction level of recipients of the Pfizer vaccine showed that most 248 (66.1%) diabetics and 175 (46.7%) non-diabetics were satisfied, whereas 92 (24.5%) diabetics and 86 (22.9%) non-diabetics were highly satisfied with the Pfizer vaccine, with a significant relationship between them (p < 0.001). No patients, either diabetic or non-diabetic, exhibited dissatisfaction with the vaccine; however, a few of them had no opinion, as shown in Table 4.

**Table 4:** The overall satisfaction with the Pfizer vaccine *p-value is significant at a <0.05.

Variable		n(%)		p-value
		Diabetes mellitus		
		Yes	No	
Overall subject level of Satisfaction for vaccine	Highly Satisfied	92(24.5%)	86(22.9%)	<0.001*
	Satisfied	248(66.1%)	175(46.7%)	
	No opinion	35(9.3%)	114(30.4%)	
	Dissatisfied	0(0.0%)	0(0.0%)	

## Discussion

Our study evaluated the association of Pfizer vaccine side effects with the presence of DM. We found that the relative frequency of Pfizer vaccine side effects was higher in diabetic patients. Moreover, overall, the vaccine side effects were minor and self-limiting. 

A systematic study assessed the side effects of the Pfizer BioNTech vaccine by examining previous research. Approximately 10,632 people participated in the 14 trials and were examined for adverse reactions to the Pfizer-BioNTech COVID-19 vaccine. Injection site pain (77.3%), fatigue (43%), muscular pain (39.67%), swelling at the injection site (33.5%), headaches (33.2%), joint pain (25.7%), chills (18.3%), fever (18%), and itching (9.38%) were typical adverse effects reported in 14 trials. After the first dose, the side effects were, on average, 79%, whereas after the second dose, they were 84% [16]. The current study was not comparable with the above-reported research and revealed that the most frequent local side effects were injection site burning: 251 (66.9%) in diabetic patients and 254 (67.7%) in non-diabetics. Additionally, muscle pain was observed in 248 (66.1%) diabetics and 153 (40.8%) non-diabetics, followed by fever in 242 (64.5%) diabetics and 283 (75.5%) non-diabetics, and injection site pain in 235 (62.7%) diabetics and 190 (50.7%) non-diabetics, following the first dose of the Pfizer vaccine. Similarly, following the second dose, the most commonly reported side effects in recipients were muscle pain, found in 240 (64.0%) diabetic patients and 194 (51.7%) non-diabetics.

Similarly, according to a study comparing the two vaccine dosages, there were more local side effects following the second injection [17]. The present study is unmatched by the said research, as the most common adverse events following the first dose of the Pfizer vaccine in both diabetic and non-diabetic participants had a higher incidence of side effects than those following the second dose of the Pfizer vaccine.

In another cross-sectional study conducted among health care workers in Iraq, there were 2,202 participants in all; 89.97% of them received COVID-19 injections, while 10.03% of them were fearful about getting the shot. The Pfizer vaccine (62.9%), AstraZeneca vaccine (23.5%), and Sinopharm vaccine (13.6%) were administered to the participants. Compared with the first dose, most side effects were substantially less common in the second dose. Interestingly, females experienced the side effects of the Pfizer vaccine at a considerably higher rate than males. The AstraZeneca vaccination had more side effects in the subjects after the first dose. Moreover, more side effects were linked to the Pfizer vaccine after the second dose [18]. The present study was inconsistent with the findings of the above-referred study and reported that out of 750 Pfizer vaccine recipients, 289 (77.1%) males and 86 (22.9%) females had diabetes, whereas 217 (57.9%) males and 158 (42.1%) females were non-diabetics. However, the adverse events following the first dose of the Pfizer vaccine were reported to be more reduced, followed by the second dose, which was inconsistent with the results of the above study. 

Abu-Hammad et al. [19] reported a higher frequency of adverse effects with the second dosage. Elnaem et al. [20] discovered that following the second dosage, nearly 40% of side effects were more common, mostly in individuals who received the Pfizer vaccine compared with those who received the Sinovac or AstraZeneca vaccine. The abovementioned study did not support this study because the local and systemic side effects in the recipients were more frequent and increased after the first dose.

While most studies stated that persons with DM were less likely than healthy individuals to develop serious side effects after the COVID-19 vaccination, Lee et al. indicated that people with diabetes had a greater likelihood of grade 3 to 4 adverse events. Pain, redness, and swelling at the injection site are the most frequent local side effects, whereas headache, chills, fever, and tiredness are the most frequent systemic side effects. After immunization, most side effects are minor, disappear in a few days, and have no impact on regular activities. Not a single patient died, and even those with new-onset DM or hyperglycemia experienced prompt resolution of their symptoms with appropriate care [21]. The present study was inconsistent with the above-reported study and revealed that the most frequent general adverse effects in both diabetes and non-diabetes patients were fever, muscle pain, and joint pain, whereas pain, burning, and swelling at the injection site were the localized side effects, followed by the first and second doses of the Pfizer vaccine. The presence of comorbidities, such as DM, significantly increased the probability of side effects after receiving the Pfizer vaccine. 

A study of the Arab region by Alghamdi et al. [22] revealed that chronic disorders are associated with the development of post-vaccination side effects. However, more research is needed to elucidate the reasons for the augmented side effects in comorbid patients receiving different COVID-19 vaccines. In the current study, participants associated with comorbidities such as DM and hypertensive patients, with a mean duration of 4.03 ± 3.12 years with DM and four years with non-diabetes, were at higher risk of receiving post-vaccination side effects of the Pfizer vaccine.

Another cross-sectional study was conducted at a multi-center in Karachi (Pakistan) and revealed that 187 (34.6%) diabetic patients received the Pfizer vaccine and 234 (38.4%) non-diabetic recipients received Sinovac. After the first dose, adverse events were highly frequent in diabetic patients; burning (injection site), pain, and fever were the most frequently occurring side effects following the first dose of the Pfizer vaccine. Similarly, after administration of the second dose, burning (injection site), muscular pain (myalgia), fever, and pain (injection site) were the most common side effects, with a significant relationship between diabetics and non-diabetics (p < 0.001) [23]. In our study, burning (injection site) and muscular pain (myalgia) were the most frequently observed side effects after receiving the first dose of the vaccine. Additionally, muscular pain and rashes as general side effects and pain at the injection site were the most commonly reported local side effects in patients with diabetes after the second dose. 

This research has some limitations. Because this was an observational study, participants with side effects that were not clinically assessed and might have been related to factors other than the vaccine were used, making it impossible to establish the causality of serious incidents, as advised by the WHO. More research is required to identify significant side effects and establish a direct causal relationship. Furthermore, our study did not compare the long-term effects of vaccination or vaccine-induced immunity with those of natural immunity following infection.

## Conclusions

This study concluded that participants with DM had considerably more frequent local and general adverse effects than those without diabetes. The most commonly observed adverse effects in both diabetic and non-diabetic participants were burning at the injection site, rashes, muscle pain, and fever after receiving the first and second doses of the Pfizer vaccine. These side effects were transitory, self-limited, and non-injurious to public health. Most recipients were satisfied with the Pfizer vaccine. Consequently, more studies are needed to improve the results and avoid spreading negative information about COVID-19 vaccinations.
